# The role of the mycobacterial DNA-binding protein 1 (MDP1) from *Mycobacterium bovis* BCG in host cell interaction

**DOI:** 10.1186/1471-2180-12-165

**Published:** 2012-08-03

**Authors:** Ralph Kunisch, Elisabeth Kamal, Astrid Lewin

**Affiliations:** 1 Division 16 Mycology/Parasitology/Intracellular Pathogens, Robert Koch Institute, Nordufer 20, 13353, Berlin, Germany

**Keywords:** *Mycobacterium*, Tuberculosis, Mycobacterial DNA-binding protein 1, MDP1, DNA-binding protein, Histone-like protein, Latency, Granuloma, Virulence, Host interaction

## Abstract

**Background:**

*Mycobacterium tuberculosis* differs from most pathogens in its ability to multiply inside monocytes and to persist during long periods of time within granuloma in a status of latency. A class of proteins called mycobacterial histone-like proteins has been associated with regulation of replication and latency, but their precise role in the infection process has yet to be uncovered. Our study aimed at defining the impact of the histone-like protein MDP1 from *M. bovis* BCG (mycobacterial DNA-binding protein 1, corresponding to Rv2986c from *M. tuberculosis*) on early steps of infection.

**Results:**

Previously, a BCG (Bacillus Calmette Guérin) strain had been generated by antisense-technique exhibiting reduced MDP1 expression. This strain was now used to analyse the impact of reduced amount of MDP1 on the interaction with human blood monocytes, macrophage lines and PBMC (peripheral blood mononuclear cells). MDP1 was revealed to be required for growth at acidic pH and for intracellular replication in human blood monocytes. Down-regulation of MDP1 resulted in reduced secretion of the cytokine IL-1β by infected human PBMC. In addition, a reduction of MDP1 expression had a major impact on the formation of fused multi-nucleated macrophages. In monocyte preparations from human blood as well as in human and mouse macrophage cell lines, both the percentage of multi-nucleated cells and the number of nuclei per cell were much enhanced when the monocytes were infected with BCG expressing less MDP1.

**Conclusion:**

MDP1 from *M. bovis* BCG affects the growth at acidic pH and the intracellular replication in human monocytes. It furthermore affects cytokine secretion by host cells, and the formation of fused multi-nucleated macrophages. Our results suggest an important role of MDP1 in persistent infection.

## Background

*Mycobacterium tuberculosis* is one of the leading causes of death due to a single infectious agent. Its success is based on perfect adaptation to the human host and the conditions prevailing in infected cells and tissues such as hypoxia, nutrient starvation, low pH and the presence of antimicrobial substances. By adapting their gene expression, growth and metabolism to these environmental conditions, the bacteria are able to persist over long periods of time inside immune cells within granuloma in a latent state until possible reactivation and outbreak of disease. To be able to combat the disease, it is necessary to understand the molecular mechanisms regulating mycobacterial intracellular persistence, latency and reactivation.

A class of proteins implicated in regulating latency are the mycobacterial histone-like proteins (Hlp) [1]. Hlp have been identified in pathogenic as well as environmental mycobacteria [2,3]. Proteins belonging to this class have been given different designations in different mycobacterial species such as HLP_Mt_ or HupB in *M. tuberculosis*[3,4], MDP1 (mycobacterial DNA-binding protein 1) in *Mycobacterium bovis* BCG [5], Hlp in *Mycobacterium smegmatis*[2] and ML-LBP21 in *Mycobacterium leprae*[6]. They are composed of an extremely basic C-terminal part homologous to eukaryotic histone H1 and an N-terminal region similar to HU from *Escherichia coli*[3,5]. Hlp expression is developmentally regulated and up-regulation was observed in dormant *M. smegmatis*[2] and stationary cultures from *M. bovis* BCG [5]. It is an immunogenic protein detectable in tuberculosis patients [7]. Hlp are nucleoid-associated proteins [8], but have also been found to be surface-exposed [5,6,9,10]. They bind to DNA [3,5] preferring AT-rich DNA-sequences [11] as well as to laminin, hyaluronic acid, heparin, and chondroitin sulphate [5,6,12]. The data available so far portray Hlp as multi-faceted proteins, and accordingly a variety of possible functions have been ascribed to Hlp. Hlp were suggested to impact DNA packaging, protection of DNA from enzymatic and non-enzymatic strand breakage [11], gene regulation [1], nucleic acid metabolism, non-homologous-end-joining repair [13], adaptation to hypoxic conditions [2], induction of dormancy [2], adaptation to cold shock [14], adhesion [6,9,12,15-17], cell wall biogenesis [10] and regulation of growth rate [1,5,10]. A role in transition to the non-culturable state and in resuscitation from the non-culturable state was shown in *M. smegmatis*[18]. Whiteford et al. [19] investigated the growth characteristics of an *M. smegmatis* with a deletion of *hlp*. They found that the mutant showed less aggregation in broth cultures. Furthermore, they observed an increased sensitivity towards Isoniazid. The *M. smegmatis* mutant also was affected in UV-resistance and resistance towards freezing/thawing. Takatsuka et al. [20] have recently shown that Hlp has a similar activity to ferritin superfamily proteins and protects DNA by ferroxidase activity. It furthermore captures iron molecules and functions as iron storage protein.

Approaches to elucidate the functions of Hlp by mutagenesis did not always confirm the expected roles of Hlp [2,15,21]. Our own attempts to generate a MDP1 deletion mutant had failed. Furthermore and in line with our own experience, Sassetti et al. [22] had shown by high density mutagenesis that the gene Rv2986c from *M. tuberculosis,* which is homologous to MDP1 from BCG, is required for optimal growth of *M. tuberculosis*. We therefore followed the strategy to analyse Hlp functions by down-regulation of Hlp expression by antisense-technique. Advantages of this technique are the possibility to analyse essential genes and to repress genes present in several copies. In mycobacteria the antisense-technique has been applied to down-regulate ahpC from *M. bovis*[23], dnaA from *M. smegmatis*[24], FAP-P from *M. avium* subsp. *paratuberculosis*[25] or pknF from *M. tuberculosis*[26]. In a previous study we described the generation of the antisense-strain *M. bovis* BCG (pAS-MDP1) which carries the plasmid pAS-MDP1 causing a reduction of MDP1 expression in BCG by about 50% [27]. We analysed BCG (pAS-MDP1) with respect to general growth characteristics. The down-regulated BCG grew faster in broth culture and achieved a higher cell mass in the stationary phase. Similarly, growth was enhanced in human and murine macrophage-like cell lines. A further important finding was the reduced protein synthesis occurring under hypoxic conditions [27]. These findings support a role of MDP1 in growth regulation of *M. bovis* BCG. Since we postulate the growth rate to be of major importance for virulence and intracellular persistence, we now investigated the role of MDP1 for the interaction of BCG with host cells during early phases of infection.

## Results

### MDP1 is essential for adaptation of BCG to low pH

**Figure 1:**
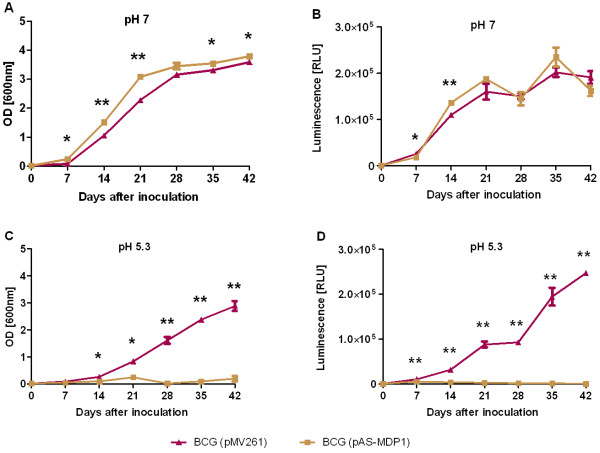
**Growth in acidic medium.** BCG (pMV261) and BCG (pAS-MDP1) were grown in Mb/OADC medium adjusted to pH 7 (A, B) or pH 5.3 (C, D), respectively, and the growth of the mycobacteria was monitored by measurement of the OD [600 nm] (A, C) and the amount of ATP in the cultures (B, D). The ATP amount was measured using a luminescence assay and is reported as relative light units (RLU). Each value represents the mean of three cultures with the standard deviation. The results of a paired student’s t test are shown by asterisks (*: P < 0.05, **: P < 0.01).

Bacteria present in activated macrophages have to face low phagosomal pH conditions. We therefore tested the ability to adapt to low pH of *M. bovis* BCG, containing the empty cloning vector pMV261 [BCG (pMV261)], and of *M. bovis* BCG with the MDP1-antisense-plasmid pAS-MDP1 [BCG (pAS-MDP1)], by comparing the growth without and with pH stress. Bacteria were grown to optical density (OD) 3 [600 nm], then diluted and inoculated into fresh Middlebrook 7H9 (Mb) /Oleic Acid-Dextrose-Catalase (OADC) medium adjusted to pH 7 and pH 5.3, respectively, and growth was monitored by measurement of OD and ATP content. As shown in Figure 1A, BCG (pAS-MDP1) reached a slightly higher OD in medium with neutral pH in comparison to BCG (pMV261) and also a higher maximal amount of ATP (Figure 1B). In medium adjusted to pH 5.3 only BCG (pMV261) was able to grow (Figure 1C, D). The growth rate of BCG (pMV261) in low pH medium was slightly below its growth rate in neutral medium if determined by OD measurement. In medium adjusted to pH 7 BCG (pMV261) grew to an OD of 3.6 after 42 days (Figure 1A). In contrast the OD of cultures grown in medium adjusted to pH 5.3 was only 2.9 after 42 days (Figure 1C). The strain BCG (pAS-MDP1) behaved very differently at low pH. It was not able to adapt to the low pH conditions and showed no growth at pH 5.3 (Figure 1C, D).

### MDP1 plays a role in persistence of BCG in human blood monocytes

**Figure 2:**
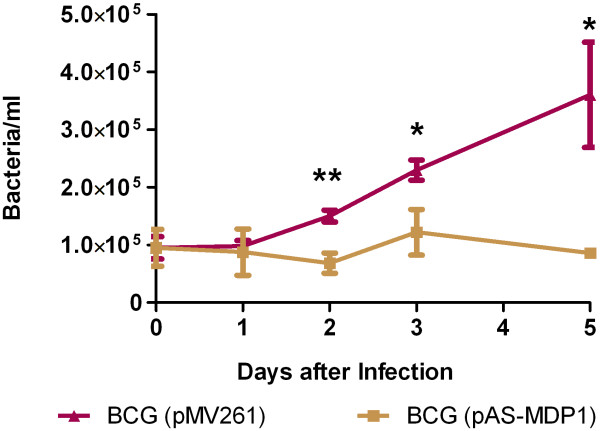
**Intracellular survival.** Human blood monocytes were infected with BCG (pMV261) and BCG (pAS-MDP1) at an MOI of 1, and the amount of intracellular bacteria in the cell lysates was determined by real-time PCR. The values represent the mean of three wells with the standard deviation. The results of a paired student’s t test are represented by asterisks (*: P < 0.05, **: P < 0.01).

The alveolar macrophages represent the first line of defence the mycobacteria have to overcome in order to establish a successful long-lasting infection. We therefore analysed the ability of our BCG strains to survive in human blood monocytes. Monocytes were infected with BCG (pMV261) and BCG (pAS-MDP1) grown to OD 2 at an MOI of 1 and the amount of intracellular bacteria was quantified one, two, three and five days after infection by quantitative real-time PCR. As shown in Figure 2, the BCG with the empty plasmid started multiplying after one day post infection. After five days, 3.8 times more cells of BCG (pMV261) were present than after the initial infection period of four hours. In contrast, the BCG containing the MDP1-antisense-plasmid stayed relatively constant throughout the whole experiment. It only showed little growth between days two and three and otherwise decreased in number. MDP1 thus plays an important role for survival and growth of BCG in monocytes.

### MDP1 affects the cytokine secretion of infected PBMC

**Figure 3:**
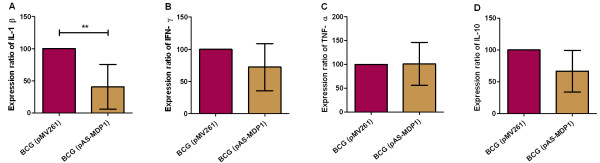
**Cytokine secretion by human PBMC.** Human PBMC were infected with BCG (pMV261) and BCG (pAS-MDP1) at an MOI of 1, and the amount of IL-1β (**A**), IFN-γ (**B**), TNF-α (**C**) and IL-10 (**D**) in the supernatants was quantified by ELISA 24 hours after infection. The values were referred to the amount of cytokines induced by BCG (pMV261), which were set to 100%. The columns represent the mean of at least five independent experiments (different donors) with the standard deviation. The results of an unpaired student’s t test showing the significance of different expressions in PBMC infected with BCG (pMV261) and BCG (pAS-MDP1) are represented by asterisks (**: P < 0.01).

The immune response against mycobacterial infections is coordinated by cytokines, and we therefore investigated cytokine expression of human PBMC induced by infection with BCG (pMV261) compared to BCG (pAS-MDP1). The PBMC were infected with the two strains at an MOI of 1 and the amount of selected pro- and anti-inflammatory cytokines (IFN-γ, TNF-α, IL-1β, IL-10) present in the supernatants was measured after 24 hours. Negative controls consisted of uninfected cells, and positive controls were activated with LPS and IFN-γ. All cytokines were induced upon activation with LPS/IFN-γ and upon infection with mycobacteria (data not shown). As shown in Figure 3, the down-regulation of MDP1 resulted in a decreased secretion of IL-1β (n = 7 donors), IFN-γ (n = 5), and IL-10 (n = 5). However, if means from all donors were calculated, only the reduction in IL-1β secretion was statistically significant (Figure 3A). The amount of IL-1β in supernatants of PBMC infected with BCG (pAS-MDP1) was only 41% of that in supernatants of PBMC infected with BCG (pMV261). No effect was observed on the secretion of TNF-α (Figure 3C).

### MDP1 influences the rate of macrophage fusion

Since the fusion of macrophages and the formation of multi-nucleated cells is one of the hallmarks of chronic infections associated with granuloma formation [28] we were interested in analysing the effect of MDP1 on macrophage fusion. To this end we infected the mouse macrophage line RAW264.7, the human macrophage line Mono Mac 6 (MM6) and monocytes isolated from human blood with BCG (pMV261) and BCG (pAS-MDP1). Uninfected cells served as negative controls and cells activated with LPS and IFN-γ as positive controls. At different times after infection (up to 11 days) the cells were stained with Diff Quick and the nuclei per cell were counted to determine the fusion index (FI). Ziehl-Neelsen staining was performed to confirm uptake of mycobacteria by multi-nucleated cells (data not shown).

**Figure 4:**
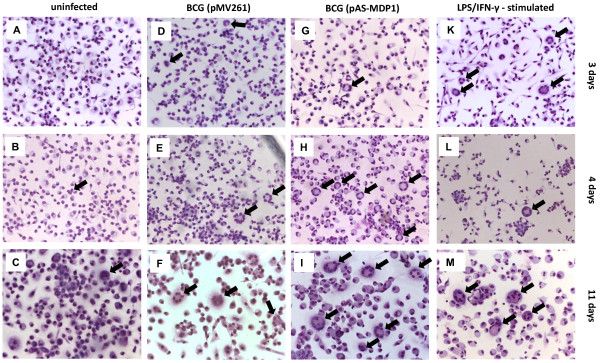
**Formation of multi-nucleated cells by human blood monocytes.** Monocytes were isolated from human blood and infected with BCG (pMV261) (**D**, **E**, **F**) or BCG (pAS-MDP1) (**G**, **H**, **I**), respectively. Uninfected cells (**A**, **B**, **C**) served as negative control. Blood monocytes activated with LPS and IFN-γ are shown in **K**, **L**, **M**. The cells were stained with Diff-Quick after three (**A**, **D**, **G**, **K**), four (**B**, **E**, **H**, **L**) and 11 (**C**, **F**, **I**, **M**) days. Micrographs were taken with a magnification of 200 ×. Arrows mark multi-nucleated cells.

The time course of fusion of human blood monocytes is shown in Figure 4. In uninfected human blood monocytes, very few multi-nucleated cells were present only after four days (Figure 4A, B), while the infected cells and the positive controls had fused already at day three (Figure 4D, G, K). At day four, clear differences were visible between the different experimental settings (Figure 4B, E, H, L). The uninfected control had formed only very few fused cells with only three nuclei (Figure 4B), while the infected cells had produced more fused macrophages with a much higher number of nuclei (Figure 4E, H). In Figure 4E [infection with BCG (pMV261)], for example, up to nine nuclei per cell are visible, and in Figure 4H [infection with BCG (pAS-MDP1)] up to 12 nuclei per cell can be counted. At this time point the LPS/IFN-γ-stimulated blood monocytes had also formed fused cells, but additionally cell aggregates were formed, which were not visible in the other experimental settings (Figure 4L). Eleven days after infection cells had enlarged, and with the exception of the negative control the fusion process had proceeded. The fusion indexes of blood monocytes 11 days after infection are shown in Table 1. The BCG strain down-regulated with respect to MDP1 expression depicted a fusion index of 15.1% which was 1.7 times higher than the fusion index induced by BCG with the empty vector pMV261 (8.7%). Especially at early time points most of the nuclei were arranged in a circle at the outer rim of the monocytes and depicted the morphology typical of the Langhans cells present in tuberculous lesions [29].

**Table 1:** Fusion index of different macrophages/monocytes after infection with BCG (pMV261) and BCG (pAS-MDP1)

**Cell type**	**MOI**^**a**^	**Days after infection**	**Fusion index (FI) [%]**		
			**Uninfected cells**	**Infection with BCG (pMV261)**	**Infection with BCG (pAS-MDP1)**
RAW264.7	50	5	3.0	5.3	27.2
MM6	50	3	2.3	2.3	7.4
Human blood monocytes	1	11	1.1	8.7	15.1

^a^ MOI = multiplicity of infection (number of mycobacteria per number of monocytes/macrophages).

**Figure 5:**
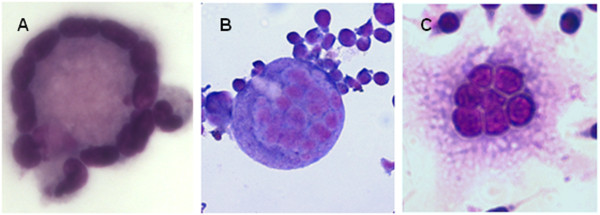
**Morphology of multi-nucleated cells.** Human blood monocytes (**A**), MM6 cells (**B**) and RAW264.7 cells (**C**) were infected with BCG (pAS-MDP1) and stained with Diff-Quick. Micrographs were taken with a magnification of 400 × .

The fusion process in the macrophage cell lines RAW264.7 and MM6 was followed over a shorter time of up to five days, because of experimental limitations due to multiplication of the cells. The results obtained with primary human blood monocytes could be confirmed by the use of the two cell lines. As shown in Table 1 RAW264.7 infected with BCG (pAS-MDP1) had formed 5.1 times more multi-nucleated cells after five days than RAW264.7 infected with BCG (pMV261). The cell line MM6 presented 3.2 times more multi-nucleated cells after infection with BCG (pAS-MDP1) than after infection with the reference strain three days after infection (Table 1). The different cell types varied with respect to maximal fusion indexes reached. Upon infection with BCG (pAS-MDP1), for example, RAW264.7 achieved the highest fusion index with 27.2% followed by human blood monocytes with 15.1%. The lowest fusion activity was observed with MM6 cells that only reached a fusion index of 7.4% (Table 1). The different types of monocytes furthermore differed with respect to the morphology of the fused cells (Figure 5). The morphology typical of Langhans cells characterised by nuclei arranged in a circle along of the periphery of the cell was only present in human blood monocytes (Figure 5A). RAW264.7 cells were shaped more irregularly, and the nuclei were concentrated in the central part of the cells (Figure 5C). Multi-nucleated MM6 cells were strongly enlarged, round, and the nuclei were spread relatively evenly across the cells (Figure 5B).

**Figure 6:**
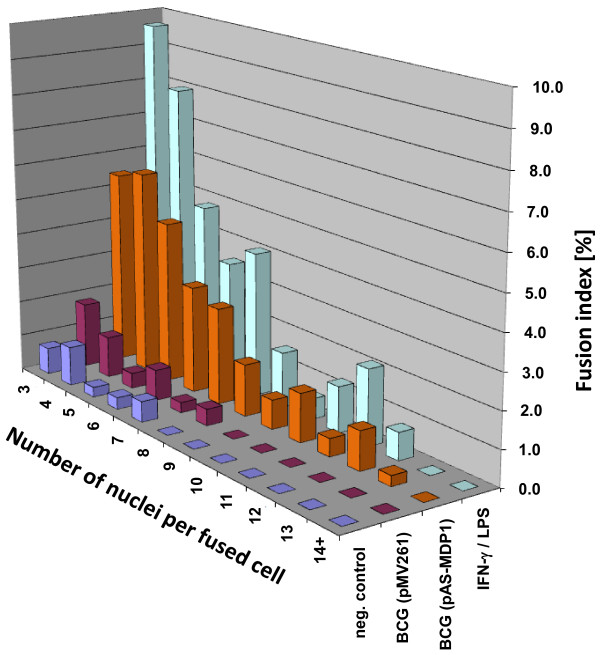
**Number of nuclei in multi-nucleated RAW264.7 cells.** RAW264.7 cells were infected with BCG (pMV261) and BCG (pAS-MDP1) at an MOI 50. Uninfected cells served as negative controls and cells activated with LPS and IFN-γ served as positive controls. Five days after infection the cells were stained with Diff-Quick, and the nuclei per multi-nucleated cells were counted and the fusion indexes calculated.

The fusion process then was analysed in-depth by calculating the fusion indexes with respect to the number of nuclei per cell. Figure 6 is a graphic illustration of the distribution of the fusion indexes in the cell line RAW264.7. The uninfected cells generated multi-nucleated cells up to only seven nuclei per cell. Up to eight nuclei per fused cell were present in RAW264.7 infected with BCG (pMV261). Much more fused cells with much higher numbers of nuclei were present in the LPS/IFN-γ-activated cells as well as in cells infected with BCG (pAS-MDP1). The highest number of nuclei per cell was found in cells infected with BCG (pAS-MDP1) with 13 nuclei per fused macrophage. From this illustration it is obvious that the fusion rates of strain BCG (pMV261) were more similar to those of uninfected cells, while the fusion rates of strain BCG (pAS-MDP1) resembled more those of cells activated with LPS and IFN-γ.

## Discussion

A large number of different functions have been proposed for the MDP1 protein from *M. bovis* BCG, but its role in infection has not been fully elucidated so far. To better understand its role in infection, we investigated its influence in very early stages of infection, and gave particular attention to its interactions with blood-derived immune cells. Our studies were performed with a BCG strain down-regulated with respect to expression of MDP1 by antisense-technique [BCG (pAS-MDP1)] and a control strain containing the empty vector without antisense-construct [BCG (pMV261)]. By using BCG (pMV261) as control, we have ensured that the tested strain and the control strain only differ by the presence of the antisense-sequence. Different reactions of the two strains can therefore be attributed to the antisense-sequence. This is supported by our experiments with other BCG genes and antisense-sequences also cloned into pMV261, which generated different results depending on the inserted sequence (data not shown). It therefore can be concluded that the inserted sequences and not the vector or additional RNA accumulation are responsible for the differing phenotypes of control and test strains.

When mycobacteria are ingested into and reside in macrophages, they are exposed to an environment characterised by decreasing pH from around 6.4 in resting macrophages to around 5.2 in activated macrophages and below 5.0 in phagolysosomes [30-33]. Accordingly we started by investigating the resistance to low pH of our two strains. The growth was monitored in broth adjusted to either pH 7 or 5.3, the latter corresponding to the pH present in activated macrophages. Although BCG (pAS-MDP1) grew better at pH 7 than BCG (pMV261), the reduction of the MDP1 protein caused an inability of these mycobacteria to adapt to low pH, resulting in complete absence of growth at pH 5.3 (Figure 1C, D).

This remarkable sensitivity towards low pH of BCG down-regulated in MDP1 expression might be an obstacle for an intra-phagosomal lifestyle, and we consequently investigated intracellular growth of the two strains in human blood-derived monocytes. We quantified intracellular BCG by real-time PCR, because we found this method more precise than colony counting. On the one hand, DNA quantification is not that much affected by clumping of BCG and presence of viable but non-culturable cells, on the other hand this method bears the risk of including dead bacteria. In a study of Barrera and colleagues [34], it was, however, shown that quantification of growth of intracellular BCG within macrophages during four days by a PCR method yielded results equivalent to those obtained by cfu counting or measurement of uracil incorporation. Again, the BCG (pAS-MDP1) showed no growth while BCG (pMV261) was able to multiply inside the monocytes (Figure 2). MDP1 thus plays a major role in intracellular survival, perhaps by enabling the bacteria to adapt to conditions present in the phagosomes such as low pH. Further experiments such as blocking of phagosome acidification or investigation of the presence and function of vATPase in the phagosome membrane are required to confirm a functional relationship between the influence of MDP1 on low pH tolerance and intracellular replication. In previous experiments, using cell lines (J774A.1 and MM6) instead of primary blood cells, we had made observations diverging from the results reported in this study. In those cell lines the MDP1-down-regulated strain showed better growth than the control strain [27]. There are several possible explanations for the different outcomes of infections of the cell lines versus blood monocytes. One plausible explanation is that primary monocytes on the one hand and cell lines on the other hand dispose of very different properties. It was shown that cell lines such as MM6, U-937 or THP-1 correspond to immature monocytes expressing biochemical markers characteristic of immature cells in monocyte development, which are not expressed by peripheral blood monocytes. Correspondingly, markers expressed at high levels in mature monocytes (e.g. lysozyme, CD14, MHC class II) were not expressed or expressed at low levels in these cell lines [35]. Deregulation of immune signalling may also occur in cell lines. The cell line J774A.1, for instance, continuously synthesizes IL-1β (ATTC product description). Such properties may affect the mycobactericidal activity of cell lines compared to primary blood monocytes. In contrast to the cell line cultures which consisted of only one cell type, namely the MM6 or J774A.1 cells, our blood monocyte preparations which were purified by Ficoll/Percoll gradient centrifugation contained about 70% monocytes and about 30% CD14-negative cells (data not shown). The latter fraction contained cells such as CD4-positive IFN-γ-secreting lymphocytes able to activate monocytes. An activation of the primary monocytes by IFN-γ-producing cells may have intensified the bactericidal activity of blood-derived monocytes compared to the cell lines.

Infection with *M. bovis* BCG (pAS-MDP1) caused a lesser activation of PBMC than infection with BCG (pMV261), as is evident from the cytokine expression of infected PBMC: 24 hours after infection the pro-inflammatory cytokine IL1-β was secreted at significantly lower amounts upon infection with the antisense-strain (Figure 3). IFN-γ as well as the anti-inflammatory cytokine IL-10 were also secreted at lower amounts, but due to donor variation no significance was obtained for the latter cytokines if the mean of all donors was calculated. The expression of these cytokines is mediated via binding of pathogen molecules to Toll-like receptors (TLR) located on the plasma and/or phagosome membranes. Among the TLR, TLR2, TLR9 and TLR4 are responsible for recognising *M. tuberculosis*. TLR4 is activated by heat shock proteins 60/65 [36,37]. The heterodimers TLR2/TLR1 and TLR2/TLR6 can recognise mycobacterial lipoproteins. TLR2/TLR1 also bind mycobacterial cell wall glycolipids including lipoarabinomannan, lipomannan and phosphatidylinositol mannoside [38] and a role of MDP1 in cell wall synthesis has been proposed by Katsube et al. [10]. CpG containing DNA represents the ligand of TLR9 [39]. An influence of MDP1 on TLR9 signalling has been shown by Matsumoto [40] who proved that addition of MDP1 protein to CpG DNA enhances the TLR9-dependent immune stimulation of this DNA resulting in increased synthesis of pro-inflammatory cytokines. The latter finding is in line with our observation of reduced synthesis of pro-inflammatory cytokines after infection with the MDP1 down-regulated BCG. In addition to TLR, the cytosolic nucleotide-binding and oligomerisation domain-like receptors such as NOD1 and NOD2 are able to bind pathogen ligands and activate cytokine expression through NF-κB. Signalling through NOD2 seems to require intracellular metabolically active bacteria [39]. Therefore, reduced cytokine secretion upon infection with the MDP1-antisense-strain may also be related to the reduced intracellular growth of this strain.

The interplay of cytokines secreted upon infection with *Mycobacterium* effects an attraction of immune cells to the site of infection, finally ending in the formation of granuloma. Multi-nucleated macrophages [multi-nucleated giant cells (MGC) also called Langhans cells] resulting from macrophage fusion reside in the middle of these structures and are considered to be hallmarks of granuloma. MGC are unable to phagocytose additional mycobacteria due to decreased expression of phagocytosis receptors. Their role seems to be to destroy mycobacteria that have been ingested by less differentiated macrophages and monocytes and to present mycobacterial antigens [41]. Lay et al. [41] showed that the maximal number of nuclei per fused macrophage depended on the infecting mycobacterial species, although all tested mycobacteria were able to induce granuloma formation. *M. tuberculosis* was able to induce MGC containing 15 and more nuclei per cell, while less pathogenic or opportunistic mycobacteria such as *M. bovis* BCG, *M. microtiM. aviumM. kansasiiM. smegmatis* or *M. phlei* only induced the formation of multi-nucleated cells containing up to seven nuclei per cell. We therefore wanted to find out whether MDP1 played a role in fusion of infected macrophages and had an influence on the differentiation of macrophages. Our experiments showed that in all cell types tested, including human blood monocytes as well as cell lines such as MM6 and RAW264.7, the BCG expressing less MDP1 induced a much higher rate of macrophage fusion (Table 1). In RAW264.7, for example, we counted up to eight nuclei per fused cell upon infection with BCG containing the empty vector pMV261 (Figure 6). This result is very similar to the results of Lay et al. [41] who reported that BCG-infected human macrophages contained up to seven nuclei per fused cell. When we infected RAW264.7 with BCG (pAS-MDP1), we observed up to 13 nuclei per cell, which is close to the 15 nuclei per cell reported by Lay et al. [41] to occur upon infection of human cells with virulent *M. tuberculosis*. Lay and colleagues have related lack of the chromosomal regions including the RD1 region in *M. bovis* BCG and *M. microti* compared to *M. tuberculosis* to their reduced MGC-inducing ability. Our results clearly show that MDP1 also plays a role in MGC formation.

## Conclusion

Multiple functions have been assigned to the MDP1 protein, but its precise role during the infection process has yet to be determined. We have investigated the influence of MDP1 on early events of infection. MDP1 was revealed to be crucial for adaptation to low pH, intracellular multiplication, induction of cytokine secretion and induction of macrophage fusion with generation of multi-nucleated Langhans cells. The latter being the hallmark of granuloma and chronic infection, our results support an important role of MDP1 in persistent infection.

## Methods

### Bacterial strains, media and growth conditions

The construction of the BCG Copenhagen strain BCG (pAS-MDP1) as well as the reference strain BCG (pMV261) has been described in Lewin et al. [27]. The plasmid pAS-MDP1 contains a 113 bp fragment of BCG-DNA, covering the first 102 bp of the coding sequence from the MDP1 gene and 11 bp of the untranslated upstream region with the Shine-Dalgarno sequence. The fragment was inserted into the vector pMV261 [42] downstream from the hsp60-promoter in antisense-orientation. If compared to BCG containing the empty vector pMV261 the expression of MDP1 is reduced by about 50% in BCG (pAS-MDP1) grown in broth culture [27]. Media and growth conditions have been described before [27].

### Cell lines and blood cells

The mouse macrophage cell line RAW264.7 (ATCC no TIB-71™) was maintained by passaging twice weekly in RPMI medium (Gibco®) supplemented with 10% FCS (foetal calf serum) (Biochrom). Cultivation of cells was performed in Falcon^TM^ 75 cm^2^ flasks at 37°C and with 5% CO_2_. The human macrophage cell line Mono Mac 6 (MM6, DSMZ no ACC 124) was maintained in RPMI medium supplemented with 10% FCS, 2 mM of L-glutamine (PAA), non-essential amino acids (PAA) and 1 mM of sodium pyruvate (PAA) and passaged twice a week. PBMC and blood monocytes were isolated from buffy coats from healthy, female, anonymous donors. Buffy coats were supplied by the German Red Cross which previously had obtained the donors’ consent for use of their blood donation for scientific purposes. PBMC were isolated by Ficoll-Paque^TM^ Plus (GE Healthcare) gradient centrifugation according to the manufacturer’s recommendations. After the Ficoll gradient centrifugation, the PBMC were washed twice with PBS (140 mM of NaCl, 16 mM of Na_2_HPO_4_, 2 mM of KH_2_PO_4_, 3.75 mM of KCl, pH 7.4) and resuspended in IMDM medium (PAA) with 3% human AB serum (PAA). For isolation of blood monocytes, a gradient centrifugation with Percoll^TM^ (GE Healthcare) was performed directly after the Ficoll gradient centrifugation. The monocytes were then washed twice with PBS and resuspended in IMDM medium with 3% human AB serum. To further enrich monocytes, the cells were allowed to adhere overnight and non-adherent cells were removed by rinsing. The percentage of monocytes was evaluated by quantification of the CD14^+^ population by FACS analysis using a mouse anti-human CD14 antibody (monoclonal antibody MEM-18, Immuno Tools) and a goat anti-mouse FITC-conjugated secondary antibody (Immuno Tools). A mouse IgG1 control (monoclonal antibody 203, Immuno Tools) was included to assess non-specific antibody binding. FACS analysis was performed using the BD FACSCalibur cytometer (BD Biosciences) and identified ~70% of the cell preparation as monocytes.

### Measurement of pH-resistance

Comparison of the growth rates of *M. bovis* BCG (pAS-MDP1) and *M. bovis* BCG (pMV2161) was carried out by inoculating Middlebrook 7H9 medium (pH 7) as well as 7H9 medium adjusted to pH 5.3, both containing 10% OADC and 25 μg ml^-1^ of Kanamycin. To prepare the acidic medium, we first dissolved 7H9 powder in water, then adjusted the pH to 5.3 with HCl, filter-sterilised the medium and finally added 10% OADC. Pre-cultures of both strains were first grown in Middlebrook 7H9 medium (pH 7) with 10% OADC to an OD [600 nm] of 3, and aliquots of these pre-cultures were inoculated into pH-adjusted media to obtain an initial OD of 0.02 to 0.04. Growth of the strains was monitored during 42 days by OD measurement and ATP quantification using the BacTiter-Glo^TM^ Microbial Cell Viability Assay Kit (Promega) as described in Lewin et al. [43]. This kit quantifies the number of metabolically active viable bacterial cells.

### Measurement of cytokine secretion by infected PBMC

One million (mio) PBMC per 500 μl of IMDM with 3% human AB serum were seeded into 24-well plates (Techno Plastic Products AG) together with 1 mio mycobacteria grown to OD 3. After 24 hours the supernatants were removed and frozen at −20°C until the quantification of the amounts of IFN-γ, IL-1β, IL-10 and TNF-α was performed by ELISA with the Ready-SET-Go kits from eBioscience. Negative controls consisted of uninfected PBMC. Positive controls consisted of PBMC that had been activated by addition of 10 ng ml^-1^ of LPS (from *E. coli*, Sigma Aldrich) and 100 U of IFN-γ (eBioscience).

### Measurement of intracellular persistence of BCG-derivatives in human blood monocytes

After isolation of human blood monocytes by Ficoll/Percoll gradient centrifugation, 1 mio cells in 1 ml of IMDM with 3% human AB serum were seeded into the wells of 24-well plates and allowed to adhere overnight. Non-adherent cells were then removed by rinsing and fresh medium was added to the adherent cells. Infection took place after 15 hours. BCG-strains grown to OD 2 were added at an MOI of 1, and the plates were centrifuged at 400 g for 5 min. After an infection time of four hours, extracellular BCG were removed by washing twice with IMDM and extracellular bacteria still present were killed by incubation in IMDM with 3% human AB serum and 200 μg ml^-1^ of Amikacin for two hours. The cells were then again washed twice and incubated for the rest of the experiment in medium containing 2 μg ml^-1^ of Amikacin. Samples for quantification of intracellular bacteria were taken four hours after addition of the bacteria (initial infection rate) and then every 24 hours during five days. Samples were taken by removing the supernatants, adding of 500 μl of water to the wells and incubating the plates for 15 min at 37°C for lysis of the macrophages and release of intracellular mycobacteria. The lysates were stored at −20°C. DNA was isolated from the lysates as described before [43]. We then quantified the BCG DNA as described in [43] by TaqMan-PCR amplifying a fragment of 130 bp from the 85B antigen gene using the primers MY85B FW/BW (5’-TCAGGGGATGGGGCCTAG-3′ and 5′-GCTTGGGGATCTGCTGCGTA-3′; [44]) and the dually labelled detector probe 5′-(FAM)-TCGAGTGACCCGGCATGGGAGCGT-3′-(TAMRA) [45]. The primers and the probe are specific for mycobacteria. The amount of DNA was determined by means of a standard established with known amounts of genomic BCG DNA and converted into bacterial numbers on the basis of the molecular weight of one BCG genome.

### Measurement of fusion rates of infected macrophage cell lines and blood monocytes

The induction of the fusion of macrophages by infection with the BCG-derivatives was investigated with the mouse macrophage line RAW264.7, the human macrophage line MM6 and monocytes isolated from human blood. The human monocytes were infected at an MOI of only 1, because they reacted more sensitive to BCG compared to the cell lines, which very well tolerated an infection at an MOI 50. 24-well plates were loaded with glass cover slips that had been treated as follows: the cover slips were incubated overnight in 250 ml of H_2_O with 0.5 ml of 100% acetic acid. After rinsing twice with water, they were rinsed with 95% methanol, dried at 37°C overnight and autoclaved.

5 × 10^4^ RAW264.7 cells in 1 ml of RPMI medium with 10% FCS were infected with 2.5 × 10^6^ BCG cells (MOI 50). The plates were centrifuged for 5 min at 400 g and incubated for four hours. Removal and killing of extracellular bacteria was performed as described above. After five days the cells were stained as described below.

Human blood monocytes were isolated as described above, 1 × 10^6^ monocytes were infected with 1 × 10^6^ BCG (MOI 1) for four hours, and extracellular bacteria were removed and killed as described. Staining of the cells was performed three, four and 11 days after infection.

A different procedure had to be followed for determination of the fusion rates of MM6 cells, because these cells grow in suspension. 5 × 10^6^ MM6 cells together with 2.5 × 10^8^ BCG (MOI 50) were mixed in 5 ml of RPMI with 10% FCS in Falcon tubes and incubated at 37°C and 5% CO_2_ for four hours. The tubes were gently shaken in between. Then, 35 ml of RPMI with 10% FCS were added and the tubes were centrifuged at 600 rpm for 10 min. The pellets were resuspended in 40 ml of RPMI with 10% FCS and centrifuged again. The bacteria were then resuspended in 30 ml of MM6 medium, and 200 μg ml^-1^ of Amikacin were added for two hours to kill extracellular mycobacteria. The cells were centrifuged as above, resuspended in 30 ml of RPMI with 10 FCS, centrifuged again and the pellets were finally resuspended in 10 ml of MM6 medium. 2 × 10^5^ cells in 1 ml of MM6 medium with 3 μg ml^-1^ of Amikacin were given into the wells with the cover slips. For negative controls, all three types of macrophages were incubated without bacteria. Positive controls consisted of uninfected macrophages activated with 100 U of IFN-γ (human IFN-γ: eBioscience; mouse- IFN-γ: Invitrogen) and 10 ng ml^-1^ of LPS (Sigma). Staining of the monocytes to visualise the nuclei was performed with the Diff Quick Stain Set from Medion Diagnostics. The preparations were evaluated by microscopy (Zeiss Axioskop 40), photographed (Axiocam HRc, Axiovision Rel.4.8.2), and the numbers of nuclei per monocyte were counted. Macrophages containing at least three nuclei were considered as multi-nucleated. At least 1000 nuclei were counted per preparation and the number of nuclei present in multi-nucleated cells was determined. The fusion index (FI) was calculated using the formula:

(1) $$FI\left[\%\right]=\frac{number\;of\;nuclei\;in\;multi-nucleated\;cells}{total\;number\;of\;nuclei}\times 100$$

## Competing interests

The authors declare that they have no competing interests.

## Authors’ contributions

AL designed and supervised the project and wrote the manuscript together with RK. RK and EK performed the experiments. All authors read and approved the final manuscript.
